# Regulation of ACSL4-Catalyzed Lipid Peroxidation Process Resists Cisplatin Ototoxicity

**DOI:** 10.1155/2022/3080263

**Published:** 2022-03-20

**Authors:** Feinan He, Xiaotong Huang, Guokun Wei, Xiaorong Lin, Weijian Zhang, Wei Zhuang, Wuhui He, Ting Zhan, Hai Hu, Haidi Yang

**Affiliations:** ^1^Department of Otolaryngology, Sun Yat-Sen Memorial Hospital, Sun Yat-Sen University, Guangzhou, China; ^2^Guangdong Provincial Key Laboratory of Malignant Tumor Epigenetics and Gene Regulation, Medical Research Center, Sun Yat-Sen Memorial Hospital, Sun Yat-Sen University, Guangzhou, China; ^3^Breast Tumor Center, Sun Yat-Sen Memorial Hospital, Sun Yat-Sen University, Guangzhou, China; ^4^Phase I Clinical Trial Centre, Sun Yat-Sen Memorial Hospital, Sun Yat-Sen University, Guangzhou, China; ^5^Department of Otolaryngology, Zhujiang Hospital of Southern Medical University, Southern Medical University, Guangzhou, China; ^6^Department of Oncology, Sun Yat-Sen Memorial Hospital, Sun Yat-Sen University, Guangzhou, China; ^7^Department of Hearing and Speech Science, Xinhua College of Sun Yat-Sen University, Guangzhou, China

## Abstract

Cisplatin-induced ototoxicity is one of the common side effects during its treatment and there are no effective measures to prevent it. Our study aimed to investigate the effect of ACSL4-catalyzed lipid peroxidation on cisplatin-induced hearing loss and its possible protective mechanisms. We used a variety of cisplatin ototoxicity models, including HEI-OC1 cell line, cochlear explants, and ET4 GFP+ zebrafish. After measuring the experimental concentrations of cisplatin by CCK8 assay and immunofluorescence, respectively, we examined the levels of lipid peroxidation by MDA content, 4-HNE content, and C11-BODIPY (581/591) probe. Then, we used two ferroptosis inhibitors, FER-1, and Vit-E to protect hair cells. We found that cisplatin significantly increased the levels of lipid peroxidation and that this process can be resisted by the ferroptosis inhibitors. Afterwards, we performed metabolomic assays on the cisplatin-treated hair cells. The metabolite levels were significantly altered in the experimental group compared to the control group, and the highest degree of change was observed in the glutathione metabolic pathway and the arachidonic acid metabolic pathway. Therefore, we screened the key enzymes on the arachidonic acid metabolic pathway in the hair cells after cisplatin treatment and found that ACSL4 had the greatest regulatory value. Further, we reduced the level of lipid peroxide in hair cells by specifically inhibiting the expression of ACSL4, which protected hair cells from cisplatin damage at source. In conclusion, the lipid peroxidation process regulated by ACSL4 may be an important factor contributing to the sensitivity of hair cells to cisplatin. Inhibition of ACSL4 expression may be an effective preventive measure against cisplatin ototoxicity.

## 1. Introduction

Cisplatin (CDDP) is a platinum-based antitumor drug [1]. Its widespread use in the treatment of breast cancer, ovarian cancer, and other cancers has greatly improved the survival rate of patients [1–4]. However, this process is often accompanied by serious side effects, including nephrotoxicity and ototoxicity [5]. Ototoxicity is generally defined as the effects of drugs, chemicals, or ionizing radiation on the cochlea, vestibule, and other ear organs, causing structural and functional damage and inducing auditory dysfunction or vestibular insufficiency [6]. The ototoxicity of cisplatin is mainly manifested by the damage to inner ear hair cells, spiral ganglia, and vascular striae, as well as effects on inner ear metabolism and oxidative stress processes [6, 7]. This results in damage to cochlear receptors and corresponding nerve endings, which manifests clinically as sensorineural hearing loss [7]. Once this kind of bilateral, progressive, and irreversible hearing loss occurs, it will have a serious impact on the patient's daily communication and greatly reduce the patient's quality of life after chemotherapy [5, 8]. Unfortunately, there are no methods or drugs available to reverse the death of human hair cells and neurons [7, 8]. Therefore, a safe and effective drug or method for cisplatin-induced ototoxicity early prevention may be needed for patients on a cisplatin regimen to minimize the incidence of hearing loss and improve their quality of life.

Until now, the specific mechanism of cisplatin ototoxicity has not been fully defined. Inflammatory response, ROS production, endoplasmic reticulum stress, and DNA damage have been reported as one of the mechanisms that cause the occurrence of hair cell death [9–13]. In previous studies, it was usually assumed that the cell damage caused by cisplatin eventually manifests as apoptosis, which has been confirmed by numerous studies on apoptosis-related pathways [8, 14–17]. However, inhibition of these pathways failed to completely reverse cisplatin damage to auditory cells and the resulting hearing impairment [8]. Therefore, the form of hair cell death under cisplatin damage still needs to be further investigated.

Ferroptosis is a new form of cell death that differs from necrosis and apoptosis [18, 19]. It is mainly characterized by the accumulation of ferrous ions and the accumulation of peroxidized lipids on the membrane [19–22]. It was officially included as one of the regulated cell death types by the cell death nomenclature committee in 2018 [18]. The results of our preliminary experiments suggest that ferroptosis is one of the important molecular mechanisms of cisplatin-induced hearing loss [23]. In cellular experiments, HEI-OC1 cell lines treated with cisplatin showed significantly higher levels of lipid peroxide and a significant decrease in cell survival [23]. However, the specific mechanism of ferroptosis in the occurrence and development of cisplatin ototoxicity still needs further in-depth study.

Ferroptosis begins with the Fenton reaction of free intracellular iron with hydrogen peroxide and catalyzes the generation of hydroxyl radicals, leading to the peroxidation of polyunsaturated fatty acids (PUFA) on phospholipid membranes [21, 24, 25]. These are mainly arachidonic acid and phosphatidylethanolamine, causing elevated membrane instability and ultimately cell death [21, 22]. The classical regulation of ferroptosis relies on the neutralization of lipid peroxidation by glutathione peroxidase 4 (GPX4) [21, 26–28]. Cysteine is directly supplied for glutathione synthesis via the Xc-system transporting extracellular cystine or the transsulfuration pathway [21, 27, 28]. It also promotes the recycling of GPX4 through oxidative, reduced glutathione conversion assisted by NADPH [21, 26].

Lipid peroxide accumulation is often used as a criterion to detect the occurrence of ferroptosis, as it plays a key role of “lethal factor” [24–26]. Recent studies on antiferroptosis have focused on modulating the expression of glutathione metabolism and its related pathways to enhance the neutralization of lipid peroxide and ultimately delay the development of ferroptosis [29]. Few studies have been conducted to reduce lipid peroxide production at the source, and its effects still need to be further explored.

Our study aims to discuss the specific role of the arachidonic acid metabolic pathway as a lipid peroxide production pathway in the mechanism of cisplatin-induced ototoxicity. The protective effect on hair cells was observed by modulating this pathway, providing an experimental basis for new targets of intervention in the prevention of hearing loss after cisplatin chemotherapy at the metabolic aspect.

## 2. Materials and Methods

### 2.1. Cell Culture and Drug Treatments

HEI-OC1 cells (kindly provided by F. Kalinec at the House Ear Institute, Los Angeles, CA, USA) were cultured in high-glucose Dulbecco's Modified Eagle's Medium (DMEM, Gibco BRL, Grand Island, NY, USA), supplemented with 10% fetal bovine serum (FBS, Gibco BRL, Grand Island, NY, USA) in acceptable conditions (33°C, 5% CO2) without antibiotics. For cisplatin toxicity test, HEI-OC1 cells were exposed to cisplatin at indicated concentrations for indicated hours for cell viability analysis. The HEI-OC1 cells were pretreated with different agents and then exposed to cisplatin at 20 *μ*M for 24 h.

### 2.2. Materials

We used cisplatin (CDDP, Selleck, S1166, Huston, TX, USA), ferrostatin-1 (FER-1, Selleck, S7243, Huston, TX, USA), vitamin E (Vit-E, Target Mol, T1648, Shanghai, China), and rosiglitazone (ROSI, Selleck, S2556, Huston, TX, USA). They were initially dissolved in DMSO and diluted in the culture medium (DMEM supplemented with 10% FBS) to a final concentration.

### 2.3. Organ Culture of Cochlear Explant

The method of cochlear explant removal and cultivation was described in our previous study [11]. The explants were exposed to 50 *μ*M cisplatin for 30 h after pretreatment with different agents for 24 h. The protocol was approved by the Animal Research Committee at Sun Yat-sen University.

### 2.4. Measurement of Cell Viability

HEI-OC1 cells were seeded at a density of 5000 cells/well in 96-well plates in three replicates and incubated overnight. After treatment, CCK8 (K1018, APExBio, USA) was added to each well for 2 h. The optical density (OD) values were measured at 450 nm using the Multiskan MK3 microplate reader (Labsystems, USA).

### 2.5. Assay of MDA

The cellular levels of malondialdehyde (MDA) were assessed using the corresponding assay kit (A003, Nanjing Jiancheng Bioengineering Institute, China). MDA can be condensed with thiobarbituric acid to form a red compound with a maximum absorption peak at 532 nm (Janero, 1990). The extracting solution was then added, and the cells were lysed via ultrasonication. The homogenates were incubated with the working solution in a 95°C water bath for 40 min, cooled with flowing water, and centrifuged at 4000 rpm for 10 min using an Eppendorf Centrifuge 5427 R. Then, the supernatant was transferred to 96-well culture plates and the absorbance was detected at 530 nm.

### 2.6. Assay of 4-HNE

4-hydroxynonenal (4-HNE) is an important by-product of lipid peroxidation and is widely used as a lipid peroxidation marker. Cells were blocked by incubating with bovine serum albumin (BSA). Then, the cells were incubated with anti-4-HNE at 4°C overnight. After washing with PBS, the cells were incubated with FITC or TIRTC-labeled secondary antibody for 2 h at 37°C in the dark. Fluorescence images were collected by using Olympus BX63 fluorescence microscope, and the results were analyzed using ImageJ software (NIH, Bethesda, MD, USA).

### 2.7. Lipid ROS Detection

Cells were seeded in 6-well plates and treated in acceptable conditions. After different treatments, cells were stained with 2 *μ*M C11-BODIPY (581/591) probe (Invitrogen, USA) in accordance with the manufacturer's instructions. Cells were visualized under Olympus BX63 fluorescence microscope and then analyzed by ImageJ software (NIH, Bethesda, MD, USA). Oxidized BODIPY (O-BODIPY) and reduced BODIPY (R-BODIPY) were observed at excitation/emission wavelengths of 488/510 nm (traditional FITC filter set) and 581/591 nm (Texas Red1filter set).

### 2.8. Immunofluorescence Staining

For hair cell observation in cochlear explants, we used a 60x oil-immersion lens on the Olympus BX63 fluorescence microscope and the ImageJ software (NIH, Bethesda, MD, USA) to analyze the results. The images of the cochlear explants were collected in groupings of apex, middle, and base.

### 2.9. Protein Extraction and Western Blot

The protein extraction and western blot were described in our previous study [30]. The specific primary antibodies are as follows: rabbit anti-ACSL4 (A6826, 1 : 1000, ABclonal, Wuhan, China), rabbit anti-LPCAT3 (A17604, 1 : 1000, ABclonal, Wuhan, China), rabbit anti-LOXs (A11504, 1 : 1000, ABclonal, Wuhan, China), rabbit anti-POR (YN1836, 1 : 1000, Immunoway, Plano, TX, USA), and mouse anti-GAPDH (RM2002, 1 : 10000, Beijing Ray Antibody Biotech, Beijing, China). The protein signals were detected after using the chemiluminescent substrate kit (Millipore, Darmstadt, Germany) and then analyzed by ImageJ software (NIH, Bethesda, MD, USA). Each experiment was repeated three times, and all protein expression was normalized to that of GAPDH.

### 2.10. Zebrafish Breeding

Zebrafish embryos of the ET4 transgenic wildtype hair cells that are specifically labeled produced adult fish and maintained at a density of 50 embryos per 100 mm Petri dish in 28.5°C embryo medium (15.0 mM NaCl, 0.5 mM KCl, 1.0 mM CaCl2, 1.0 mM MgSO4, 0.14 mM KH2PO4, 0.06 mM Na2HPO4, and 0.5 mM NaHCO3).

### 2.11. Lateral Line Hair Cell Counting in Zebrafish

The experiments used five days postfertilization (dpf) zebrafish. Zebrafish were placed in a plate with medium, with 8 zebrafish per hole and each group set up 2 holes. After 6 h CDDP exposure, all of them were selected to fix in 4% paraformaldehyde for 30 min. The samples were washed and made into slides for observation under Olympus BX63 fluorescence microscope. The average cell count in each zebrafish lateral line was calculated.

### 2.12. Metabolism Analysis

For understanding the metabolic environment of hair cells after cisplatin treatment, metabolites were collected according to the methods used in previous studies [31]. 80% methanol and 80% acetonitrile containing 0.02 ppm of L-Methionine-(*methyl*-^13^C,d_3_) were prepared separately. On liquid nitrogen, precooled methanol was added to walled HEI-OC1 cells rinsed with PBS and incubated at -80°C for 20 min to measure steady-state metabolite levels. Cells were collected on ice with a spatula after which acetonitrile was added and collected again. The supernatant was collected after centrifugation at 14,000*g* for 10 min (4°C) to extract the cell metabolites. The supernatant was dried and stored at -80°C and then subjected to LC-MS-/MS-based metabolite analysis. Suspend the samples in HPLC grade water. A 5500 QTRAP hybrid triple quadrupole mass spectrometer (AB/SCIEX) was utilized together with a prominence ultrafast liquid chromatography HPLC system (Shimadzu). Afterwards, analysis of a total of 95 detectable metabolites was performed by selecting reaction monitoring using positive and negative polarity switching. Total ion current peak areas were incorporated for each metabolite using MultiQuant v2.0 software (AB/SCIEX). Moreover, two parallel plates of cells were collected, the total number of cells was measured and the metabolite data were normalized.

All the metabolic data were analyzed on http://www.metaboanalyst.ca. The normalized data was uploaded, and the compound labels were standardized. The metabolite set enrichment analysis was performed using package “globaltes” based on a metabolite set library, including 99 metabolic pathway-associated metabolite sets.

### 2.13. Statistical Analysis

Statistical analyses were performed using GraphPad Prism statistical software (version 6, GraphPad Software, Inc., San Diego, CA). Comparison between two groups was analyzed by unpaired Student's *t*-test and comparison between multiple groups by one-way ANOVA. A *p* value <0.05 was considered statistically significant for all tests.

## 3. Results

### 3.1. Cisplatin-Induced Hair Cell Death Has the Characteristics of Ferroptosis

**Figure 1:**
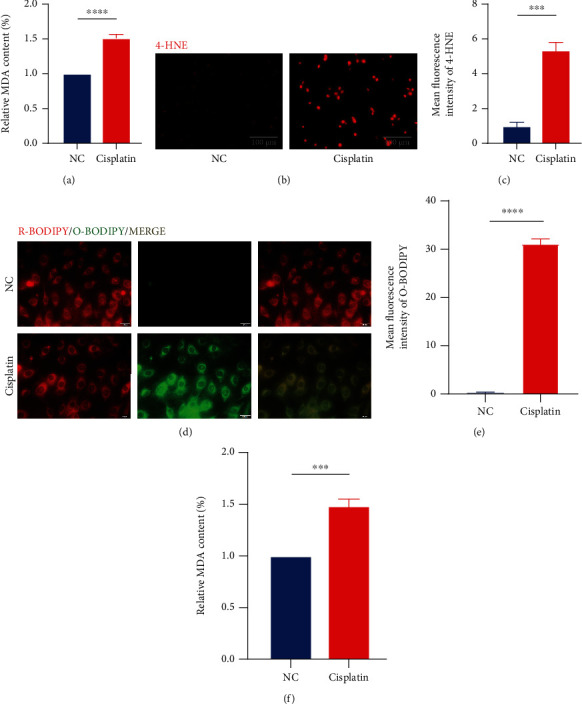
Cisplatin leads to elevated levels of lipid peroxidation in hair cells. (a) The relative MDA content levels in HEI-OC1 cells with cisplatin (20 *μ*M for 24 h) exposure. (b and c) Fluorescence images displaying 4-HNE immunostaining and the relative fluorescence intensity of 4-HNE. (d and e) Representative images of C11-BODIPY (581/591) staining in different groups of cells and the relative fluorescence intensity of O-BODIPY. (f) The relative MDA content levels in cochlear explants in cisplatin (50 *μ*M for 30 h) exposure. (The data were all quantified by ImageJ software. *n* = 3 individual experiments. The data are shown as mean ± SEM. ^∗∗∗^*p* < 0.001 and ^∗∗∗∗^*p* < 0.0001 vs the control group. NC: control group treated with same dose of DMF in cisplatin group. MDA: malondialdehyde. 4-HNE: 4-hydroxynonenal. R-BODIPY: reduced BODIPY. O-BODIPY: oxidized BODIPY.).

In order to explore the changes of lipid metabolism in hair cells after cisplatin treatment, we examined several indices of lipid peroxidation. After the treatment of HEI-OC1 cells with cisplatin, we first examined the content of MDA and 4-HNE, the products of lipid peroxidation process [8]. The results showed that the intracellular MDA content increased to approximately 1.5 times that of the control group after cisplatin treatment (Figure 1(a)). 4-HNE fluorescence intensity was also significantly increased (Figures 1(b) and 1(c)). We then used a C11-BODIPY fluorescent probe to specifically bind intracellular lipids and observed them under a fluorescent microscope [32]. The probe bound to peroxidized lipids will show green fluorescence [32]. It is quite visualized from the results that the content of lipid peroxide in hair cells is higher after cisplatin treatment than in the control group (Figures 1(d) and 1(e)).

As a further verification, we examined the MDA content in the cisplatin-treated cochlear explants of mice. Corresponding to the results of the intracellular experiments, the MDA content in the cochlear explants was significantly higher after the addition of cisplatin (Figure 1(f)).

These results suggest that cisplatin causes a significant increase in the level of lipid peroxidation in hair cells, which is one of the characteristic changes of ferroptosis.

### 3.2. Specific Inhibition of Ferroptosis Partially Protects Against Cisplatin-Induced Hair Cell Death

**Figure 2:**
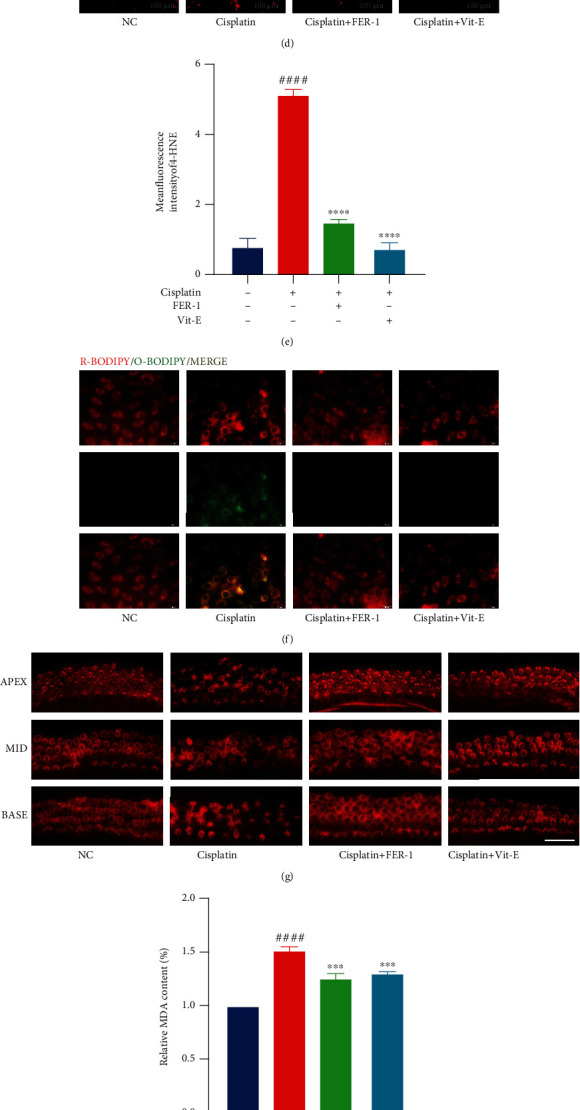
Ferrostatin-1 (FER-1) and vitamin E (Vit-E) protect hair cells from cisplatin toxicity by decreasing lipid peroxidation levels. (a and b) HEI-OC1 cells treated with varying concentrations of FER-1 and Vit-E for 24 h were analyzed by Cell Counting Kit-8 (CCK-8). (c) The relative MDA content levels in HEI-OC1 cells with cisplatin (20 *μ*M for 24 h) exposure, with or without FER-1(20 *μ*M for 24 h) or Vit-E (30 *μ*M for 24 h) pretreatment. (d and e) Fluorescence images displaying 4-HNE immunostaining and the relative fluorescence intensity of 4-HNE. (f) Representative images of C11-BODIPY (581/591) staining in different groups of cells. (g) Representative images of cochlear explants stained with phalloidin in cisplatin (50 *μ*M for 30 h) exposure, with or without FER-1(30 *μ*M for 24 h) or Vit-E (30 *μ*M for 24 h) treatment (scale bar, 20 *μ*m). (h) The relative MDA content levels in different groups of cochlear explants. (i and j) Hair cell counts and quantification obtained from zebrafish in cisplatin (400 mM for 6 h) exposure with or without FER-1(40 mM for 6 h) or Vit-E (80 mM for 6 h) treatment (scale bar, 20 *μ*m). (The data were all quantified by ImageJ software. *n* = 3 individual experiments. The data are shown as mean ± SEM. ^###^*p* < 0.001 and ^####^*p* < 0.0001 vs the control group. ^∗^*p* < 0.05, ^∗∗^*p* < 0.01, ^∗∗∗^*p* < 0.001, ^∗∗∗∗^*p* < 0.0001 and ns no significant vs the cisplatin group. NC: control group treated with same dose of DMF and DMSO in other groups. MDA: malondialdehyde. 4-HNE: 4-hydroxynonenal. R-BODIPY: reduced BODIPY. O-BODIPY: oxidized BODIPY. Apex: apical turn of cochlear base membrane. Middle: middle turn of cochlear base membrane. Base: base turn of cochlear base membrane.).

Two specific ferroptosis inhibitors, ferrostatin-1 (FER-1) and vitamin E (Vit-E), have been reported to be used [33–35]. We first examined the protective effect of FER-1 and Vit-E against cisplatin-induced cell death by CCK8 and used this to determine their optimal working concentrations (Figures 2(a) and 2(b)). These two drugs increase the survival rate of hair cells from 50% to about 80% (Figures 2(a) and 2(b)). Our results showed both FER-1 and Vit-E at concentrations of 20 *μ*M and 30 *μ*M, a statistically significant reduction in cisplatin-induced elevation of MDA and 4-HNE levels in HEI-OC1 cells, respectively (Figures 2(c)–2(e)). By observing the intensity of green fluorescence emitted by the C11-BODIPY fluorescent probe [32], we found that both drugs also significantly reduced the level of lipid peroxidation (Figure 2(f)).

In cochlear explants, we also applied both FER-1 and Vit-E, which had a significant protective effect on hair cells (Figure 2(g)). After treatment with 50 *μ*M of cisplatin for 30 hours, the inner and outer hair cells crumpled significantly or even died (Figure 2(g)). The morphological changes of hair cells were inconspicuous after the addition of FER-1 and Vit-E (Figure 2(g)). We then tested the MDA content in cochlear explants and obtained results consistent with those in the HEI-OC1 cell line, i.e., both specific ferroptosis inhibitors significantly reduced lipid peroxidation levels in cisplatin-treated cochlear explants (Figure 2(h)).

Here, we applied the ET4 GFP+ zebrafish model for further validation. The hair cells of the lateral line neuromasts of zebrafish have similar properties to those of human cochlear hair cells, making them an important tool in the field of otology and especially ototoxicity research [36–38]. Under normal conditions, there are 10-13 hair cells per neural colliculus [38], while under 600 *μ*M cisplatin treatment, only 2-4 will remain (Figures 2(i) and 2(j)). An increase in the survival rate of hair cells to about 70% was found after adding either FER-1 or Vit-E (Figures 2(i) and 2(j)).

### 3.3. Cisplatin Leads to Significant Changes in Lipid Metabolism in Hair Cells

**Figure 3:**
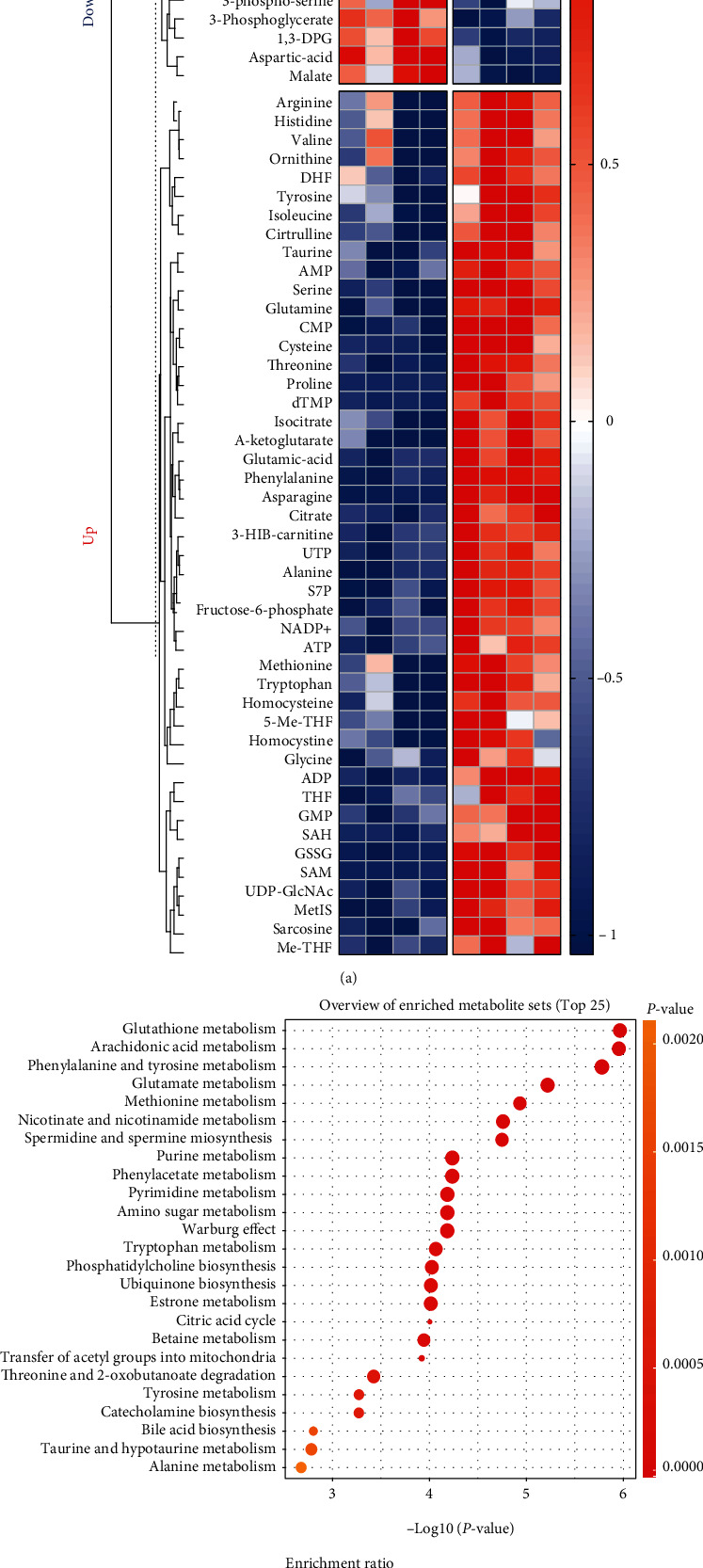
Metabolomic analysis of cisplatin-treated HEI-OC1 cells. (a) Heatmap of metabolites collected from HEI-OC1 cells with cisplatin (20 *μ*M for 12 h) exposure. (b) Overview of enriched metabolite pathways (top 25). (Analyses of data were all conducted on http://www.metaboanalyst.ca. NC: control group treated with same dose of DMF in cisplatin group.).

We conducted metabolomic testing of cisplatin-treated HEI-OC1 cells according to the methods used in previous studies [31]. Significantly different levels of metabolites were found in the cisplatin-treated group compared to the control group (Figure 3(a)). We performed pathway enrichment analysis for the significantly different metabolites and ranked the differential metabolic pathways according to the degree of enrichment (Figure 3(b)). Two pathways with the highest enrichment were glutathione and arachidonic acid metabolic pathways (Figure 3(b)).

According to previous studies, both pathways are closely related to lipid peroxidation processes [22, 26]. The glutathione metabolic pathway is the classical intracellular ferroptosis regulator [21, 27]. It relies on the neutralization of lipid peroxidation by GPX4 [27]. Direct provision of cysteine for glutathione synthesis via the Xc-system transporter extracellular cystine or the transsulfuration pathway and facilitation of GPX4 recycling through oxidative reduced glutathione conversion with the aid of NADPH [27]. This also makes GPX4 one of the key signature proteins of ferroptosis. The glutathione metabolic pathway has been most extensively studied in the mechanism of ferroptosis, and it represents a pathway for the neutralization of intracellular lipid peroxidation.

The arachidonic acid metabolic pathway, on the other hand, represents the process of lipid peroxidation production [39]. A variety of PUFA with arachidonic acid and adrenoic acid as the main substrates on the membrane structure are catalyzed by acyl coenzyme A synthetase long-chain family member 4 (ACSL4) to produce their corresponding coenzyme A [26]. And catalyzed by lysophosphatidylcholine acyltransferase 3 (LPCAT3), lipoxygenases (LOXs), and cytochrome P450 oxidoreductase (POR), peroxidized lipids are formed [26]. This leads to a gradual increase in cell membrane instability and eventual cell disintegration [26, 39]. This is the underlying source of the occurrence of ferroptosis [39].

The above alterations in metabolic pathways suggest that cisplatin-induced changes in hair cell metabolism are mainly disorders of lipid metabolism. This also implies that modulation at the level of lipid metabolism is of great significance for hair cell protection.

### 3.4. Modulation of Arachidonic Acid Metabolic Pathway Reduces Hair Cell Sensitivity to Cisplatin

Currently, most of the studies on antiferroptosis focus on oncology and other fields [20, 24]. However, most of them are focused on the regulation of glutathione metabolism, which is the most essential intracellular pathway for the direct reduction of lipid peroxide [27]. But would inhibition from the source of lipid peroxidation be more effective? On the basis of such doubts, we focused our study on exploring the arachidonic acid metabolic pathway.

**Figure 4:**
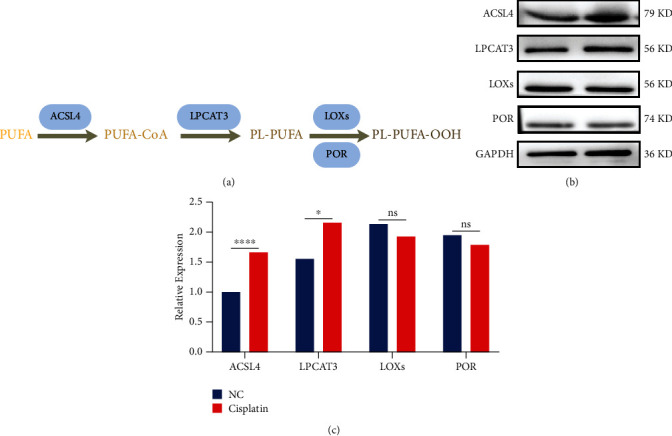
Screening of key enzymes in the arachidonic acid metabolic pathway. (a) Illustration of the arachidonic acid metabolic pathway. (b and c) Representative western blots of ASCL4, LPCAT3, LOXs, and POR expression and the quantified expression levels. (The data were all quantified by ImageJ software. *n* = 3 individual experiments. The data are shown as mean ± SEM. ^∗^*p* <0.05, ^∗∗∗∗^*p* < 0.0001, and ns no significant vs the control group. NC: control group treated with same dose of DMF and DMSO in other groups. PL: phospholipid. PUFA: polyunsaturated fatty acid. PL-PUFA: phospholipids with polyunsaturated acyl tails. ASCL4: acyl coenzyme A synthetase long-chain family member 4. LPCAT3: lysophosphatidylcholine acyltransferase 3. LOXs: lipoxygenases. POR: cytochrome P450 oxidoreductase.).

As mentioned before, there are a series of key enzymes in the arachidonic acid metabolic pathway, such as ACSL4, LPCAT3, LOXs, and POR. (Figure 4(a)) [21]. They gradually oxidize polyunsaturated fatty acids and eventually end up forming lipid peroxides (Figure 4(a)) [21, 26]. Will and how does cisplatin affect their expression in hair cells? We examined the expression of these enzymes in cisplatin-treated HEI-OC1 cells by western blotting. The expression of these enzymes was found to be varied to a greater or lesser extent compared to the control group. The increase in the expression of ACSL4, which is the rate-limiting enzyme for the initial step of the polyunsaturated fatty acid metabolism reaction, was approximately 1.7 times that of the control (Figures 4(b) and 4(c)) [40]. There was an increase in LPCAT3 expression, but to a lesser extent than in ACSL4 (Figures 4(b) and 4(c)). Slightly reduced expression was observed for LOXs and POR, but no statistical difference was found (Figures 4(b) and 4(c)).

ACSL4 showed the most significant degree of alteration and was the rate-limiting enzyme at the most initiation step. When the amount of ACSL4 was increased, its catalytic reaction rate was increased, and thus, the degree of lipid peroxidation was also increased accordingly [40]. Thus, we speculate that ACSL4 may be the most regulated of all these targets.

**Figure 5:**
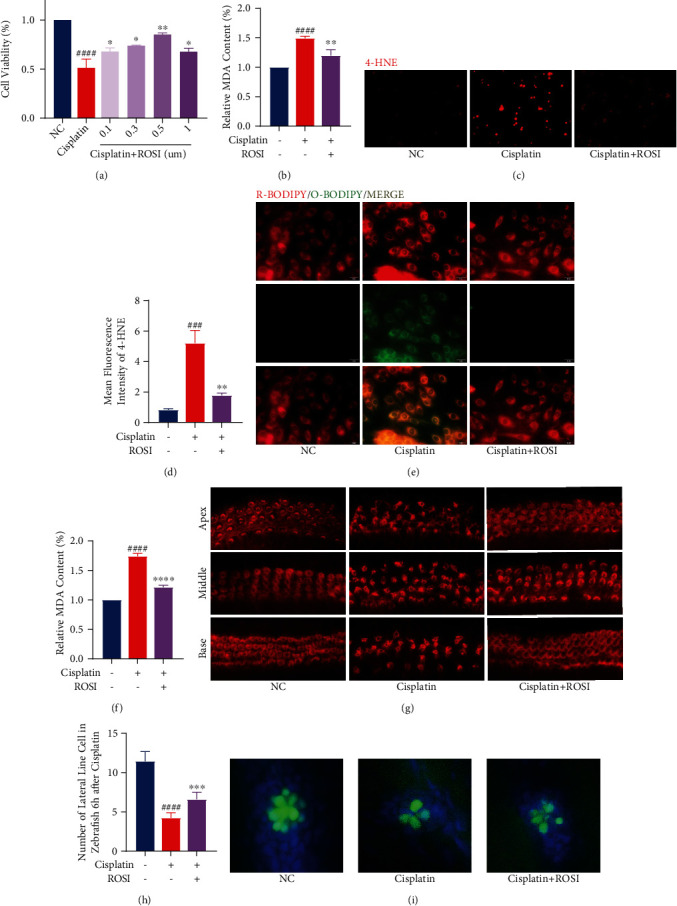
Rosiglitazone (ROSI) protects hair cells from cisplatin toxicity by decreasing lipid peroxidation levels. (a) HEI-OC1 cells treated with varying concentrations of ROSI for 24 h were analyzed by Cell Counting Kit-8 (CCK-8). (b) The relative MDA content levels in HEI-OC1 cells with cisplatin (20 *μ*M for 24 h) exposure, with or without ROSI (0.5 *μ*M for 24 h) pretreatment. (c and d) Fluorescence images displaying 4-HNE immunostaining and the relative fluorescence intensity of 4-HNE. (e) Representative images of C11-BODIPY (581/591) staining in different groups of cells. (f) Representative images of cochlear explants stained with phalloidin in cisplatin (50 *μ*M for 30 h) exposure, with or without ROSI (30 *μ*M for 24 h) treatment (scale bar, 20 *μ*m). (g) The relative MDA content levels in different groups of cochlear explants. (h and i) Hair cell counts and quantification obtained from zebrafish in cisplatin (400 mM for 6 h) exposure with or without ROSI (40 mM for 6 h) treatment (scale bar, 20 *μ*m). (The data were all quantified by ImageJ software. *n* = 3 individual experiments. The data are shown as mean ± SEM. ^###^*p* < 0.001 and ^####^*p* < 0.0001 vs the control group. ^∗^*p* < 0.05, ^∗∗^*p* < 0.01, ^∗∗∗^*p* < 0.001, and ^∗∗∗∗^*p* < 0.0001 vs the cisplatin group. NC: control group treated with same dose of DMF and DMSO in other groups. MDA: malondialdehyde. 4-HNE: 4-hydroxynonenal. R-BODIPY: reduced BODIPY. O-BODIPY: oxidized BODIPY. Apex: apical turn of cochlear base membrane. Middle: middle turn of cochlear base membrane. Base: base turn of cochlear base membrane.).

We tried to inhibit its expression using rosiglitazone (ROSI), a specific inhibitor of ACSL4 [41, 42]. The cell viability tested by CCK8 suggested that the protective effect of ROSI in cisplatin-treated HEI-OC1 cells was comparable to that of FER-1 and Vit-E (Figure 5(a)). In HEI-OC1 cells, ROSI also had a significant reduction in their lipid peroxide levels (Figure 5(b)). We can clearly see that the fluorescence intensity of 4-HNE was markedly reduced after the application of ROSI (Figures 5(c) and 5(d)), and the level of lipid peroxide was also found to be much lower using the C11-BODIPY fluorescent probe (Figure 5(e)). Similarly, ROSI had a remarkable protective effect on mouse cochlear explants (Figure 5(f)) and substantially reduced the MDA content (Figure 5(g)). In zebrafish experiments, ROSI could also provide effective protection against cisplatin ototoxicity (Figures 5(h) and 5(i)). This suggests by inhibiting ACSL4, ROSI also resists the elevated level of lipid peroxidation induced by cisplatin, i.e., it inhibits the ferroptosis process.

**Figure 6:**
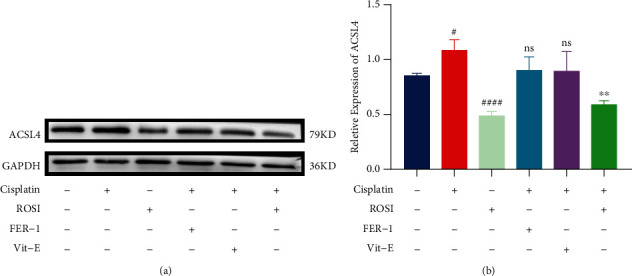
Comparison of the protective mechanism of rosiglitazone (ROSI) with ferrostatin-1 (FER-1) and vitamin E (Vit-E) against cisplatin toxicity in hair cells. (a and b) Representative western blots of ASCL4 expression and the quantified expression levels. (The data were all quantified by ImageJ software. *n* = 3 individual experiments. The data are shown as mean ± SEM. ^#^*p* < 0.05 and ^####^*p* < 0.0001 vs the control group. ^∗∗^p < 0.01 and ns no significant vs the cisplatin group. Control group treated with same dose of DMF and DMSO in other groups. ACSL4: acyl coenzyme A synthetase long-chain family member 4.).

As a further exploration of the similarities and differences between the mechanism of ROSI in hair cell antiferroptosis and the other two ferroptosis inhibitors, we coupled cisplatin with ROSI, FER-1, and Vit-E, respectively. The expression of ACSL4 was examined by western blotting. ACSL4 expression was elevated by treatment with cisplatin in hair cells (Figures 6(a) and 6(b)). When ROSI was added alone, the expression of ACSL4 was reduced to half of that of the control, suggesting that ROSI was inhibiting the function of ACSL4 by reducing the expression (Figures 6(a) and 6(b)). We found that the expression of ACSL4 in the FER-1 and Vit-E groups was similar to that of the cisplatin group (Figures 6(a) and 6(b)). This is consistent with some previous studies that FER-1 and Vit-E mainly neutralize peroxidized lipids directly without affecting the upstream processes [34, 35]. However, the expression of ACSL4 in the group of hair cells cotreated with cisplatin and ROSI was remarkably lower than that in the group with cisplatin alone (Figures 6(a) and 6(b)).

Again, this demonstrates that ACSL4 catalyzes the upstream process of lipid peroxidation production. When its expression was markedly reduced, the level of lipid peroxidation in hair cells was also reduced, and then the survival rate of hair cells was relatively much improved. By regulating ACSL4, the production of lipid peroxide could be inhibited and the sensitivity of hair cells to ferroptosis could be reduced at source satisfactorily. This is what the other two ferroptosis inhibitors cannot achieve.

## 4. Discussion

Cisplatin-induced hair cell death via ferroptosis has been reported in several early studies [23, 29, 43]. We chose to detect the process of lipid peroxidation, a hallmark alteration of ferroptosis, in which MDA and 4-HNE, as products of lipid peroxidation, can be used to detect the occurrence of ferroptosis [21, 26]. Also, C11-BODIPY fluorescent probe can be specific for lipid ROS, which is also the most common assay for ferroptosis [32]. The hair cells treated with cisplatin showed corresponding alterations in the occurrence of ferroptosis in all of these indexes.

FER-1 and Vit-E, widely used as ferroptosis inhibitors, are primarily thought to act as an antioxidant to neutralize lipid peroxidation [33–35]. Our results demonstrate their reassuring role in combating ototoxicity. This also coincides with the fact that ferroptosis is one of the mechanisms of cisplatin-induced hair cell damage. It is possible to significantly improve the survival of hair cells by specifically neutralizing peroxidized lipids.

It is also the first time that we propose to explore the changes in hair cells after cisplatin treatment from a metabolic perspective by metabolomics assays. Cisplatin-induced alterations in hair cell metabolomics were closely associated with ferroptosis [26]. In our results, it was shown that the metabolic pathways most significantly altered in cisplatin-treated hair cells were the glutathione metabolic pathway and the arachidonic acid metabolic pathway. Changes in glutathione metabolic pathways often occur in conjunction with oxidative stress processes. The relationship between the glutathione metabolic pathway and ferroptosis has been confirmed [28]. Its changes suggest that cisplatin leads to a decrease in intracellular neutralization of lipid peroxide, which exacerbates the occurrence of ferroptosis [28]. In contrast, the arachidonic acid metabolic pathway, which is a metabolic pathway for polyunsaturated fatty acids, including arachidonic acid, is upregulated in response to cisplatin, with a consequent increase in lipid peroxide production in hair cells, also promoting the development of ferroptosis [39]. Both of these two most obvious metabolic alterations are closely related to the process of the onset and development of ferroptosis.

We focused our study on the arachidonic acid metabolic pathway for the reason that it represents the process of lipid peroxidation production [39]. After screening multiple key enzymes in the arachidonic acid metabolic pathway, such as ACSL4, LPCAT3, LOXs, and POR, we found ACSL4 to be particularly critical as the key enzyme that catalyzes the initiation of the entire reaction. After ACSL4 inhibition by using ROSI, we found that the expression of ACSL4 decreased, lipid peroxide content decreased, and mortality of hair cells decreased. In contrast, when hair cells were protected with some common ferroptosis inhibitors, such as FER-1 and Vit-E, only a decrease in lipid peroxide content was observed, but no effect on ACSL4 expression [34, 35]. This suggests that ACSL4 catalyzes the initiation of lipid peroxide production. Through its intervention, lipid peroxidation can be inhibited at the source, resisting ferroptosis and reducing the damaging effects of cisplatin on hair cells, which implies a reduced sensitivity of hair cells to cisplatin. This is an effect that cannot currently be achieved with other ferroptosis inhibitors. Several previous studies have reported clinical cases of ACSL4 gene deletion with deafness [44, 45]. This also supports our speculation that ACSL4 has an important place in auditory function.

There are still some shortcomings in our study. For the effect of cisplatin on ACSL4 expression, we only observed a downregulation effect on its expression. However, by which method this effect was achieved and how it altered ACSL4 activity still remains to be further investigated. Also, we have not found a specific mechanism for the inhibitory effect of ROSI on reducing ACSL4 expression in any study so far, which needs to be thoroughly explored. We will investigate in following experiments whether ROSI can show a protective effect in the mouse model of cisplatin-induced hearing loss.

## 5. Conclusion

By promoting lipid peroxidation, cisplatin can cause ferroptosis in hair cells. This damage can be partially inhibited by specific ferroptosis inhibitors. We found that the arachidonic acid metabolic pathway and glutathione metabolic pathway play an important role in the mechanism of cisplatin injury through the detection of metabolites. ACSL4, among them, is the most essential rate-limiting enzyme catalyzing the metabolic pathway of polyunsaturated fatty acids, including arachidonic acid. By using ROSI to inhibit ACSL4 expression, hair cells can be protected from the source of lipid peroxidation. The expression of ACSL4 may also be a key factor contributing to cisplatin sensitivity in hair cells, making ACSL4 a possible novel biological target for preventing cisplatin ototoxicity as well.

## Data Availability

The data used to support the findings of this study are available from the corresponding author, Haidi Yang, upon request.
